# Mate-Finding as an Overlooked Critical Determinant of Dispersal Variation in Sexually-Reproducing Animals

**DOI:** 10.1371/journal.pone.0038091

**Published:** 2012-05-25

**Authors:** James J. Gilroy, Julie L. Lockwood

**Affiliations:** Department of Ecology, Evolution and Natural Resources, Rutgers University, New Brunswick, New Jersey, United States of America; University of California, Berkeley, United States of America

## Abstract

Dispersal is a critically important process in ecology, but robust predictive models of animal dispersal remain elusive. We identify a potentially ubiquitous component of variation in animal dispersal that has been largely overlooked until now: the influence of mate encounters on settlement probability. We use an individual-based model to simulate dispersal in sexually-reproducing organisms that follow a simple set of movement rules based on conspecific encounters, within an environment lacking spatial habitat heterogeneity. We show that dispersal distances vary dramatically with fluctuations in population density in such a model, even in the absence of variation in dispersive traits between individuals. In a simple random-walk model with promiscuous mating, dispersal distributions become increasingly ‘fat-tailed’ at low population densities due to the increasing scarcity of mates. Similar variation arises in models incorporating territoriality. In a model with polygynous mating, we show that patterns of sex-biased dispersal can even be reversed across a gradient of population density, despite underlying dispersal mechanisms remaining unchanged. We show that some widespread dispersal patterns found in nature (e.g. fat tailed distributions) can arise as a result of demographic variability in the absence of heterogeneity in dispersive traits across the population. This implies that models in which individual dispersal distances are considered to be fixed traits might be unrealistic, as dispersal distances vary widely under a single dispersal mechanism when settlement is influenced by mate encounters. Mechanistic models offer a promising means of advancing our understanding of dispersal in sexually-reproducing organisms.

## Introduction

Despite being a key determinant of population and community dynamics, dispersal is arguably the least understood of all ecological processes. Dispersal predictions are essential in forecasting population responses to environmental change, making robust models of dispersal highly desirable for biodiversity conservation [1], [2]. The distribution of dispersal events in space is critical for population dynamics, for example, determining the degree of connectivity between subpopulations or the rate of geographic spread for invasive species [3]–[5]. Accordingly, there is a prodigious literature concerning the evolution of dispersal strategy, the eco-evolutionary correlates of dispersive traits and the proximate forces that influence how dispersal behaviors are expressed [reviewed in e.g. 2,6–9]. Despite this level of interest, robust predictive models of animal dispersal remain elusive, hinting that significant gaps persist in our understanding [1], [2], [8], [9]. In this paper, we highlight one such gap: the influence of mate-finding on dispersal patterns amongst sexually-reproducing organisms.

Dispersal in animals is typically governed by a set of physiological and cognitive traits that determine how and when exploratory movements occur [1], [2]. The distance moved by a disperser is partially dependent on these traits, which we collectively refer to as the dispersal mechanism, and partially on the conditions encountered by the individual during its lifetime [8]–[11]. Understanding the mechanisms underpinning dispersal is a key frontier in ecology, but progress is hampered by the difficulty of directly observing the cognitive processes used to make dispersal decisions (e.g. emigration, movement and settlement choices). Much remains to be learned about the behavioral decision-making algorithms that underpin dispersive movements, as well as the degree of variation in dispersal patterns that arises when these algorithms are expressed in nature [1], [2].

A wide range of biotic and abiotic variables are known to influence dispersal patterns, most of which have been subject to detailed study. These include life history traits [8], [9], movement mechanisms [1], [12], [13], density-dependent competition [14]–[18], the risk of inbreeding [19], [20] and environmental conditions [21]–[23]. The role of mate-finding in influencing dispersive movements, however, has received relatively little attention in the dispersal literature [24]. Some studies have explored the ways mate availability influences the evolution of dispersal behavior, demonstrating for example that mate scarcity can drive coevolution of male and female dispersal kernels [24], [25]. To our knowledge, however, no studies have directly examined the influence of mate-finding interactions on the dispersive movements of sexually-reproducing animals [24]. In particular, little attention has been paid to the links between population density and dispersal in systems where mate-finding is an important determinant of settlement in one or both sexes.

Often, models examining dispersal make the tacit assumption that settlement decisions are based purely on the availability of habitat (e.g. [13], [21], [26], [27]). Reproductive encounters are seldom modeled explicitly, and it is usually assumed that reproduction occurs whenever a threshold number of individuals is present in a given habitat patch. In many cases, this may be a valid simplifying assumption, as the movements associated with mate-finding may operate at a smaller spatial scale than ‘true’ dispersal (i.e. within rather than between patches) [28], [29]. However, this assumption may be unrealistic at low population densities, when relatively large movements might be required in order for individuals to locate mates. We note that dispersal at low densities is particularly important for spatial population dynamics, for example determining patterns of movement at range edges and the colonization of new sites [30]–[32].

For sexually-reproducing organisms, it is intuitive that the fitness consequences of dispersal may be contingent on the availability of mates in the destination site. Consequently, we might expect sexually-reproducing individuals to directly consider mate availability as a basic requirement when making settlement decisions. If the probability of encountering a mate varies with the distribution of conspecifics across the landscape, a pervasive relationship between density and settlement probability is likely to emerge. We therefore hypothesize that mate-finding interactions represent a fundamental component of variation in dispersal distances. To evaluate this concept, we use an individual-based simulation model to examine how dispersal patterns are influenced by variations in mate-finding probability under fluctuating population size and density. By constructing simple ‘null’ models of movement behavior, we seek to re-cast the animal dispersal paradigm from first principles, placing mate availability centrally within the suite of criteria animals employ to make settlement decisions.

## Methods

### Simple Models of Active Dispersal

**Figure 1:**
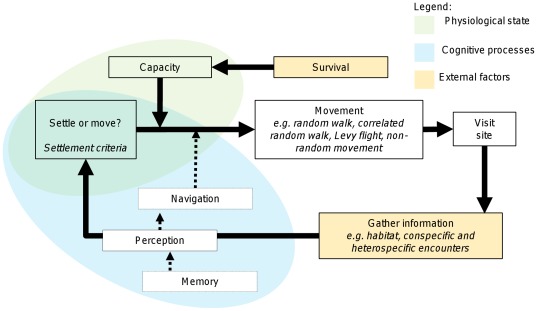
Conceptual model of active dispersal mechanisms in animals (adapted from [1]. Dispersal is viewed as a continuous process of environmental sampling where individuals assess environmental characteristics (including conspecific and heterospecific encounters) relative to a set of settlement criteria. Decision-making is potentially influenced by memory and the capacity to navigate (allowing comparative prospecting of sites), as well as the physiological state of the individual. Various mechanisms of movement between sampling events are possible, including Lévy flight where step lengths scale according to a probability distribution with a power-law tail.

The dispersal process can be viewed as a series of behavioral transitions marking an individual’s path from an initial ‘floating’ state to a temporary or permanent state of residence (e.g. a breeding site, home range, territory). This process involves interactions between the internal state of the disperser (physiological and cognitive) and the environment encountered at each step (Fig. 1). Dispersive movements themselves can be separated into two key elements: 1) the nature of movement (e.g. random walk, correlated random walk or Lévy flight [1]), and 2) the mechanism by which settlement decisions are made (e.g. threshold selection criteria or comparative prospecting [33], [34]). We envisage the resultant dispersal pathway as a sampling process in which individuals gather information across successive movements, culminating in their making some form of settlement decision [34].

We use mechanistic models to examine variability in dispersal distances in populations where individuals disperse according to simple behavioral algorithms. Individual movements are simulated within a landscape that supports realistic variations in population size and density. As a basic model with broadest possible generality, we consider a random walk movement algorithm that requires no high-order cognition or navigation [2], [13]. We simulate the dispersive movements of each individual using threshold-based settlement criteria, where an individual ceases to move (i.e. settles) as soon as a given set of threshold conditions is encountered. We examine the variation in population-scale dispersal patterns under these simple dispersal algorithms, assuming that individuals commence assessment of their environment from the moment of independence from parents.

### Model Structure

To minimize the confounding effects of environmental variation, our simulations take place within a simple landscape lacking spatial or temporal heterogeneity. In all simulated scenarios, individuals move within an unbounded two-dimensional cellular environment, where each individual can move in any direction for a distance constrained only by the time available for movement. Each cell is identical, and dispersal occurs in discrete time intervals, with each year being broken into 300 time steps. At each time step, individuals assess the cell in which they currently reside with respect to a set of settlement criteria. If the criteria are met, they remain in the cell until the next time step. If the criteria are not met, they move into an adjacent cell, with the direction of that movement drawn at random from a bounded uniform distribution (0–359.9°). Speed of movement is constant, such that the distance moved in each time step is constrained to one cell width for all individuals. For simplicity, perceptual range is limited to the cell in which the individual is located at a given time step. Settlement criteria are identical for all same-sex individuals in each scenario.

**Figure 2:**
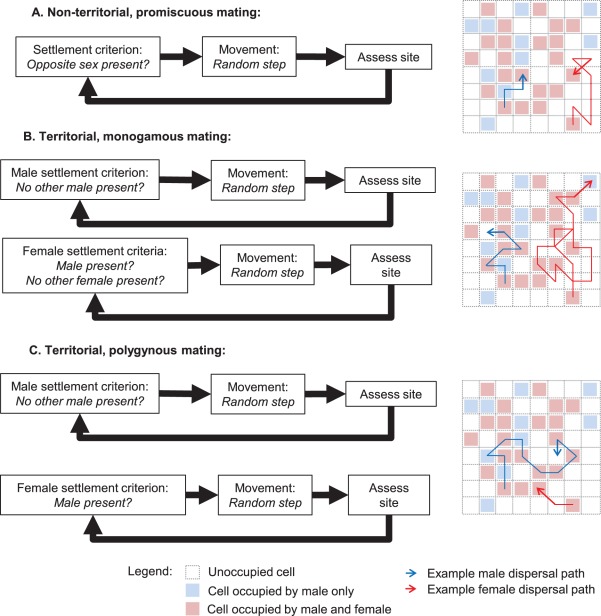
Examples of simple random walk dispersal algorithms. This schematic diagram shows the behavioral algorithms used by simulated dispersers in order to make movement and settlement decisions in our simulation models. Three scenarios are modeled, corresponding with three life history strategies: a non-territorial system with promiscuous mating, in which threshold settlement criteria are identical for both sexes (A); a monogamous territorial system where territories are established by males, and females settle once they locate an available mate (B); and a polygynous territorial system where territories are established by males, and females settle as soon as they locate a mate, regardless of the presence of other females (C). Insets show simulated examples of dispersal paths for individual males and females under each algorithm, within a small section of the model environment.

We construct models representing widespread life history strategies amongst animals, specifically considering mating system (monogamous and polygynous) and intra-specific resource competition (territorial and non-territorial). Each life history is realized in the model by varying the set of settlement criteria for each sex (Fig. 2). We thus consider three scenarios: A) non-territoriality with promiscuous mating, B) territoriality with monogamous mating and C) territoriality with polygynous mating. In scenario A, settlement criteria are identical for both sexes: the cell must simply contain at least one member of the opposite sex. As such, there is no limit to the number of individuals settling in a single habitat cell. In the territorial scenarios (B and C), single-cell ‘territories’ are established by males, which settle according to the same criterion in both scenarios: the absence of a previously-settled male. In the monogamous territorial scenario (B), female settlement is based on two criteria: the cell must contain 1) at least one male and 2) no previously settled females. The polygynous territorial scenario (C) differs in that the second criterion is relaxed for females, allowing multiple females to settle within a single male territory.

We initially seed all simulations with 500 individuals (of equal sex ratio) placed at random across the landscape, with the starting distribution constrained to a 100×100 cell central area. Individuals disperse across the landscape according to the above mechanistic rules, and reproduce when females settle in habitat cells with available males. Birth and death rates vary stochastically between years (see below), resulting in yearly fluctuations in population density across the landscape.

At the start of each year, individuals assess the cell in which they reside and either settle there if criteria are met, or begin to disperse. Dispersal proceeds for a maximum of 300 time steps, although individuals settle as soon as their criteria are met. The order in which individuals move is randomized each year. Reproduction occurs the end of the year, followed by deaths, before another year begins. As such, all individuals alive at the start of a given year are guaranteed to survive the full 300 time steps of that year (i.e. there are no fitness costs associated with dispersal). Each female that successfully settles with a mate in a given year is able to reproduce, and both fecundity and reproductive success are stochastic. Fecundity (i.e. the number of offspring *n* raised in any one breeding attempt) is drawn from a uniform distribution bounded between 1 and 4, whilst reproductive success (i.e. successful raising of *n* offspring to independence) is then determined by a Bernoulli trial with a fixed probability of 0.75. Offspring sex ratio is fixed at parity. Inbreeding avoidance is incorporated by prohibiting offspring from settling in the cell in which they were born.

In order to generate stochastic fluctuations in population size between years, annual survival probability *S_i_* is allowed to vary in a density dependent manner according to the following equation (from [35]):
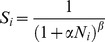
where



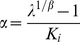
and *λ* is a parameter determining stochastic variation in survival rate, drawn from a lognormal distribution with fixed mean *μ* and standard deviation σ; *β* is a constant giving the magnitude of density dependence; *N_i_* is the population size at the start of year *i;* and *K_i_* is the carrying capacity. Values of these parameters were held constant in all simulations (*µ* = 10, σ = 10, *β* = 4 and *K* = 1,000). Survival for each individual (adults and offspring) is determined at the end of each year by Bernoulli trial with probability *S_i_*, which in practice varied between 0.09 and 0.92 (mean 0.46).

At each time step, the location of each individual is recorded as coordinates *x* and *y*. At the end of each year, we compute the linear annual dispersal distance for each individual, regardless of whether settlement was achieved. Global density (i.e. population density over the whole landscape) in a given year was calculated as the number of individuals alive during the year divided by the maximum number of cells occupied in that model run (calculated from a rectangle between the highest and lowest *x* and *y* coordinates for cells where breeding was recorded). For each scenario we conducted five replicate model runs (i.e. with different random starting positions), recording dispersal data in each case for 200 years following an initial burn-in period of 100 years. Simulations were written in Visual Basic and implemented within Microsoft Excel 2010 (Version 14.0.5128.5000).

### Statistical Analysis

We derived dispersal kernels by fitting probability density functions to combined sets of annual dispersal distances, binned across a range of population densities (bin range 0.01–0.1 individuals cell^−1^, bin width 0.005). We were particularly interested in how mate availability across the landscape influenced the tail of the dispersal kernel (i.e. determining the rate of long-distance dispersal events), measured as the level of leptokurtosis. In order to evaluate leptokurtosis, we compared the fit of models from two probability families, the negative exponential and the Weibull. The Weibull distribution has two parameters (shape parameter *k* and scale parameter *λ*). The negative exponential distribution is a special case of the Weibull with a shape parameter *k* = 1. The negative exponential is considered a null model for random walk dispersal with constant settlement probability [36]. Generally, dispersal kernels are considered ‘fat-tailed’ if the probability of settlement at large distances exceeds the predictions of a negative exponential distribution [36]. Weibull functions with shape parameter *k*<1 are more leptokurtic (‘fat-tailed’) than the negative exponential distribution, indicating a higher probability of long-distance dispersal. A value of *k*>1 indicates a less leptokurtic distribution. We therefore use *k* as an indicator of the degree of leptokurtosis in a given set of dispersal distances (binned with respect to population density). Models were fitted using a maximum-likelihood algorithm within the *fitdistr* function in R package MASS [http://rss.acs.unt.edu/Rdoc/library/MASS/html/fitdistr.html]. For each binned sample, we assessed the probability that each of the two models provided the best fit using Akaike weights (AIC_w_).

## Results

**Figure 3:**
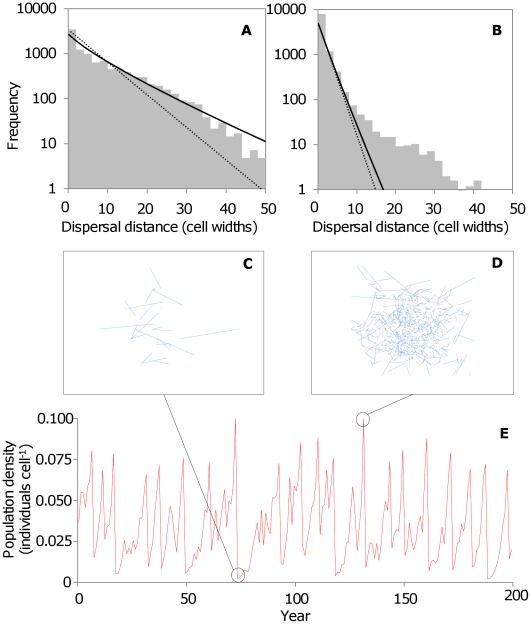
Simulations of dispersal in a simple non-territorial organism with promiscuous mating. These figures show example outputs from the promiscuity model, where individuals of both sexes settle as soon as they locate a cell containing at least one member of the opposite sex. Histograms show frequency distributions of annual dispersal distances from years with the lowest (A) and highest (B) densities, using binned data from a 300 year simulation run (excluding 100 year burn-in period; A = lowest density bin, <0.005 individuals cell^−1^; B = highest density bin, >0.095 individuals cell^−1^). Lines show negative exponential (hatched line) and Weibull (solid line) functions fitted to the data, indicating a fat-tailed distribution (Weibull *k*<1) at low densities. Insets show examples of linear dispersal movements for individual years at low (C) and high (D) density. Population density fluctuates annually due to density dependent survival, as shown in an example 200 year run following a 100 year burn-in (E).

**Figure 4:**
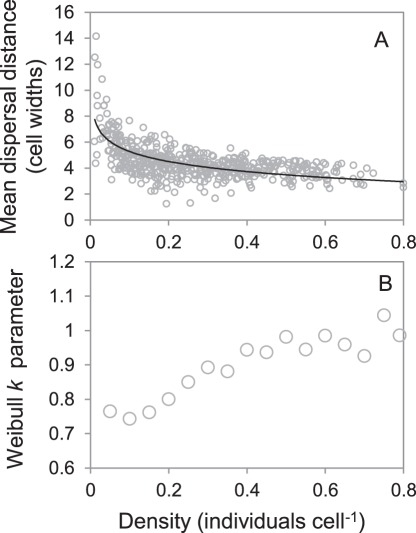
Results of simulations with promiscuous mating and no territory defense. These figures show the relationship between density and (A) mean annual linear dispersal distances, and (B) Weibull function shape parameter (*k*) values derived from binned annual dispersal distances from 1,000 years of model runs (bin size 0.005 individuals cell^−1^). When *k* = 1, the distribution conforms to a negative exponential function as expected under random walk diffusion. A value k<1 indicates increasing leptokurtosis in dispersal distances, and hence a more fat-tailed distribution.

In the promiscuous scenario where both sexes use the same settlement criteria (i.e. encountering at least one member of the opposite sex), realized dispersal distances varied markedly in relation to population density (Fig. 3). At high population densities in our promiscuous mating scenario, the realized distribution of dispersal distances approached that of a negative exponential function (Weibull *k* = 0.967 for highest bin (>0.095 individuals cell^−1^), AIC*_w_* = 1.0, Fig. 3b), indicating broad conformity with the null expectation for the dispersal kernel of diffusion under a random-walk. At low population densities, however, the dispersal kernel became strongly leptokurtic (Weibull *k* = 0.793 for lowest bin (<0.005 individuals cell^−1^), AIC*_w_* = 1.0, Fig. 3a), despite the underlying dispersal mechanism remaining unchanged. Annual population-wide mean dispersal distances showed a strong upward inflexion at population densities below 0.02 individuals cell^−1^ (Fig. 4a), whilst the degree of leptokurtosis in population-wide dispersal distances decreased linearly with increasing population density until the negative exponential distribution was approached (Fig. 4b). This pattern of leptokurtosis reflects the increased variation in search times, and hence dispersal distances, required for individuals to encounter mates when populations are small and sparsely distributed across the landscape (Fig. 3c).

**Figure 5:**
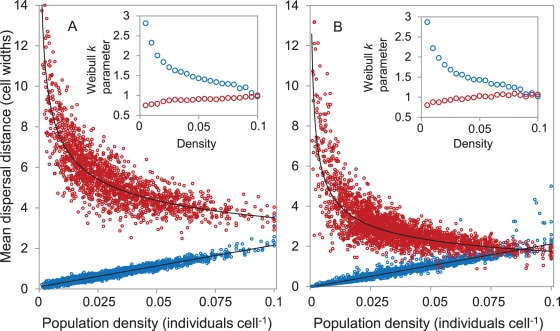
Results of simulations with male territory defense, showing the relationship between density and dispersal for monogamous (A) or polygyous (B) mating systems (1,000 year simulation runs in each case). Mean annual linear dispersal distances are shown for males (blue circles) and females (red circles) with respect to global population density. Insets show Weibull function shape parameter (*k*) values derived from binned sets of annual dispersal distances at a range of population densities (bin size 0.005). When *k* = 1, the distribution conforms to a negative exponential function as expected under random walk diffusion. A value k<1 indicates increasing leptokurtosis in dispersal distances, and hence a more fat-tailed distribution.

In scenarios involving simple territorial defense (one habitat cell per territory), dispersal patterns were also highly variable with respect to population density (Fig. 5). In the monogamous scenario with male territory defense, annual mean dispersal distances and leptokurtosis increased linearly with density (Fig. 5a), reflecting the increasing competition for free habitat cells at high population sizes. For females in this scenario, settlement depended on finding a cell with at least one male and no additional females, resulting in a sharp decrease in population-wide mean dispersal distances with density (Fig. 5a). Note that high inter-annual variance in dispersal distances at very low densities resulted from small annual samples, reflecting the low numbers of individuals surviving in those years.

In the monogamous scenario with male territoriality, female dispersal was highly leptokurtic at low population densities (Weibull *k*<0.85 for all bins <0.025 individuals cell^−1^; Fig. 5a inset) but approximated the negative exponential distribution at higher densities (Weibull *k*>0.95 for all bins >0.075 individuals cell^−1^; Fig. 5a inset). This pattern stems from the increased search times required of females to encounter unpaired territorial males when density was low. Population-wide annual mean dispersal distances of females consistently exceeded those of males, but the magnitude of sex bias was far greater at low densities (Fig. 5a). The difference in dispersal leptokurtosis between sexes disappeared at the highest densities (>1800 individuals), where dispersal of both sexes approximated the negative exponential distribution (i.e. Weibull *k* ≈ 1; Fig 5a inset). Male dispersal distances therefore increased at high densities due to competition for territories, whilst female dispersal distances decreased concomitantly due to the increased availability of mates.

In a polygynous territorial scenario, male dispersal patterns were similar to those in the monogamous scenario (Fig. 5b), as expected given that the male settlement algorithm was identical in both cases. For females, population-wide mean dispersal distances again declined with population density, but the magnitude of decline was steeper than that of the monogamous scenario (Fig. 5). There was no evidence of a secondary increase in leptokurtosis at high densities, with female dispersal consistently approximating a negative exponential distribution at densities above 0.05 individuals cell^−1^ (i.e. Weibull *k* ≈ 1; Fig. 5b inset). At the highest population sizes (all bins >1,800 individuals), the pattern of sex bias was reversed, as mean dispersal distances of males exceeded those of females in 75% of sample years (n = 28; Fig. 5b).

## Discussion

We show that simple settlement decision-rules can generate a range of different dispersal patterns at the population scale, even in the absence of spatial habitat heterogeneity or individual trait variation. When settlement decisions depend on locating a mate within suitable habitat, the probability of fulfilling settlement criteria becomes highly sensitive to variation in the abundance of conspecifics across the landscape. Surprisingly, this potentially ubiquitous source of dispersal heterogeneity has been overlooked in previous works on dispersal distance, and may be a missing component in our understanding of animal dispersal mechanisms.

Our simulations demonstrate how random walk dispersal with a single basic settlement rule can generate both fat- and thin-tailed dispersal distributions under fluctuating population density. Similarly, we show how patterns of sex-bias in dispersal can be diminished or even reversed across a gradient of population density, even when the underlying dispersal mechanisms remain unchanged. Fat-tailed kernels and sex-biased dispersal patterns are both encountered widely in nature, and have been subject to intensive study, primarily focusing on the demographic and environmental conditions in which such strategies are adaptive [6], [19], [35], [37]. Our results suggest that drawing inferences from patterns of sex-biased dispersal in wild populations may be more complicated than previously thought, as observed sex-biases might be conditional on the spatial arrangement of individuals within the population at the time of study. Evolutionary models often assume that dispersal distance itself is a trait under natural selection (e.g. [20], [32], [35], [38]–[40]); we suggest, however, that selection pressures are more likely to operate on the mechanism governing dispersal, and in particular the behavioral algorithm used to make settlement decisions [19]. Although our model did not consider evolutionary dynamics, our results highlight the variation in dispersal patterns that can arise as a result of density variation, even under the simplest of dispersal algorithms. This suggests that models treating dispersal distance as selected trait may be significantly over-simplifying the evolutionary dynamics of sexually-reproducing organisms. New insights into the evolution of dispersal may be achieved by examining how selection favors different behavioral algorithms across a range of densities, particularly in relation to the criteria used in making settlement decisions (including mate-finding) [19], [24].

Our most striking result was the substantial increase in dispersal distance exhibited at low population sizes whenever settlement was dependent on encountering a potential mate. In our territorial scenarios, the “fat-tail” of the dispersal kernel in low density conditions was not a random subsample of all individuals in the population, but was instead highly biased toward females (the sex making mate-based settlement choices in these models). This result echoes a widely-recognized example of the Allee effect, where mate scarcity is expected to limit reproductive success at low density [41], [42]. It is possible to envisage that in patchy environments, female-biased dispersal resulting from limited mate encounters could result in increasingly biased adult sex ratios in low-density patches, owing to high levels of female emigration. The role played by such sex-biased dispersal in generating Allee effects has received little previous attention (but see [24], [43]); our results suggest that mechanistic modeling of settlement decisions could generate important insights into the dynamics of animal populations at low density. In particular, our findings hint at a mechanism by which adult sex ratios might become increasingly skewed at the margins of a species’ geographical range, suggesting that the dynamics of dispersal at range edges may be more complex than traditional diffusion-based models suggest ([24], [43], Gilroy & Lockwood in prep.).

Mate-finding is likely to form just one part of a suite of factors influencing settlement decisions in any given population, and may often be swamped by the effects of habitat variability or individual trait variation. In particular, patch spacing and matrix permeability are likely to be critical in determining patterns of long-distance dispersal in organisms inhabiting patchy environments [31], [32]. Individual behavior with respect to habitat boundaries (e.g. avoidance of matrix) also plays a major part in determining long-distance movements, together with survivorship and movement rates when crossing matrix areas [44], [45]. Nevertheless, our results suggest that mate-finding interactions are likely to add a fundamental component to both spatial and temporal variation in dispersal distance, particularly in low-density settings. Our findings also highlight the difficulties implicit in using static kernel-based probability distributions to describe dispersal patterns in nature. Applications of dispersal kernels in metapopulation models, for example, make a tacit assumption that the distances moved by a sample of field-tracked individuals directly reflect fixed species or population-specific traits [46], [47]. This field-derived dispersal data is a ‘snapshot’ reflecting the dispersal patterns arising given the spatial arrangement of conspecifics within the spatial and temporal interval sampled. Our results suggest that such data may be poorly representative of patterns arising under demographic conditions that differ even marginally from those prevalent at the time of field sampling.

Given the important role of density variation illustrated here, we suggest that future empirical studies of dispersal should seek to confront the logistical difficulty of quantifying conspecific density across the landscape, allowing mate encounter probability to be estimated in a meaningful manner [48]. Continuing improvements to telemetry techniques are likely to assist greatly to this end [1]. Novel analytical approaches to dispersal modeling might seek to build on the ideal gas model [49], often used to characterize encounter rates between mobile organisms, as an approach to controlling for landscape-scale density effects [50]. We suspect that mechanistic simulation approaches, particularly combining data-based and simulation-based inference in a Bayesian framework, will be exceptionally important in advancing our understanding of animal dispersal. Mechanistic models incorporating mate-dependent settlement are likely to have increased power in explaining patterns of dispersal, particularly in sexually-reproducing organisms. Examination of complex settlement algorithms involving higher cognitive processes such as memory and navigation will also bring significant advances to our understanding of real-world dispersal patterns. These algorithms have been examined more fully in the contexts of optimal foraging and mate search behavior, but have yet to be fully explored in the context of landscape-level dispersal [24], [26], [34]. Our results suggest that these are important avenues for future study.
